# Assessing the Regulatory Functions of LncRNA SNHG11 in Gastric Cancer Cell Proliferation and Migration

**DOI:** 10.3389/fcell.2021.620476

**Published:** 2021-03-18

**Authors:** Danyi Zhao, Huawei Chen, Bing Wang

**Affiliations:** ^1^Department of Gastrointestinal Oncology, The Second Hospital of Dalian Medical University, Dalian, China; ^2^Dalian Medical University, Dalian, China

**Keywords:** apoptosis, CDC25A, gastric cancer, migration, miR-184, proliferation

## Abstract

The aim of this study was to assess the regulatory functions of SNHG11 in gastric cancer (GC) cell proliferation and migration. Dual-luciferase reporter assay and bioinformatics prediction [starBase (http://starbase.sysu.edu.cn/) and TargetScan (http://www.targetscan.org)] indicated that SNHG11 functions as a miR-184 sponge that can directly act on CDC25A. Compared with normal healthy gastric tissue and mucosal epithelial cell GES-1, SNHG11 and CDC25A expressions were dramatically increased in GC samples and cell lines, whereas microRNA-184 (miR-184) levels were reduced. SNHG11 silencing led to increased miR-184 and reduced CDC25A, whereas miR-184 downregulation recovered the expression of CDC25A. Additionally, miR-184 upregulation also played a role in regulating CDC25A ablation. Then, SNHG11 was silenced or miR-184 was upregulated in two GC cells (SGC-7901 and MKN-28). SNHG11 silencing and miR-184 upregulation caused a notable decrease in GC cell growth and proliferation and increased the apoptotic level of GC cells. Furthermore, SNHG11 silencing and miR-184 upregulation contributed to a decreased migration capacity of GC cells. Downregulated miR-184 expression in SNHG11 silenced GC cells showed that miR-184 inhibition reversed the effect of SNHG11 silencing on the growth, proliferation, apoptosis, and migration of GC cells. Moreover, *in vivo* xenograft experiments demonstrated that SNHG11 knockdown can inhibit tumor growth. These observations confirmed that SNHG11 acts as an oncogene, whereas miR-194 served as a tumor suppressor in GC development. SNHG11 may provide a new biomarker for GC diagnosis, treatment, and prognosis.

## Introduction

Gastric cancer (GC) is the fourth most prevalent cancer and the second biggest trigger of cancer-related mortality in the world (Ferlay et al., 2010). The occurrence of GC involves many steps and factors, and identifying GC subtypes will pave the way for patient stratification and targeted therapy development (Wadhwa et al., 2013; Cancer Genome Atlas Research Network, 2014). Many studies have demonstrated that the prognosis for GC molecule or protein expression profiles are probably distinct (Chen et al., 2015). Four GC molecule subtypes (Epstein–Barr virus, microsatellite instability, chromosomal instability, and genomically stable) are currently associated with different patterns of molecule changes, disease progression, and prognosis according to gene expressions (Wadhwa et al., 2013; Cancer Genome Atlas Research Network, 2014). Nonetheless, the molecular mechanisms underlying gastric occurrence are still unknown, and it is necessary to identify GC prognosis biomarkers.

An increasing amount of evidence reveals that non-coding RNAs (ncRNAs), such as microRNAs (<200 nt) and long non-coding RNAs (lncRNAs) (>200 nt) (Kolata, 2012), are associated with the occurrence of many tumors (Adams et al., 2015; Schwarzer et al., 2017). LncRNAs have been found to be highly associated with various diseases, including the occurrence, progression, and prognosis of malignancies and cardiovascular and endocrine diseases. They also play a critical role in modulating the biological processes involving cell proliferation, differentiation, and apoptosis (Pant et al., 2019; Rochet et al., 2019; Xin et al., 2019). Increasing data also suggest that lncRNAs play a role in GC development (Li et al., 2016). A previous study has confirmed that the SNHG11 expression in hepatocellular cancer (HCC) is higher. Furthermore, SNHG11 was able to promote biological processes through the modulation of microRNA-184 (miR-184) (Huang et al., 2020). Therefore, our study aims to further investigate and verify the role of lncRNA SNHG11 in GC, which may provide new insight into GC treatment.

Dysregulation of microRNAs commonly occurs in various human cancers, including GC. The tumor suppressor part of miR-184 in cancers has been widely reported. For instance, it was reported to suppress glioma cell proliferation and intrusion, as well as neuroblastoma and non-small cell lung cancer cells (Malzkorn et al., 2010; Tivnan et al., 2010; Lin et al., 2015). Nevertheless, the role it plays in GC remains unknown.

This study demonstrates the clinical influence and effects of lncRNA SNHG11 in GC, and also confirms that lncRNA SNHG11 as a competing endogenous RNA facilitates the GC malignancy properties to regulate miR-184 binding, thereby modulating CDC25A expression. These findings confirm the possibility of SNHG11 as a promising new diagnosis biomarker and treatment target.

## Materials and Methods

### Ethics Statement

The procedures conducted as part of this study were approved by the Ethics Committee of the Second Hospital of Dalian Medical University. All patients provided written informed consent before beginning of the study. Every procedure was approved by the Animal Care and Use Committee of the Second Hospital of Dalian Medical University. All possible measures were taken to decrease animal suffering as much as possible.

### Patients

A total of 33 patients with GC (average age, 55.5 ± 7.3 years) and seven healthy volunteers were enrolled from the Second Hospital of Dalian Medical University (Table 1). All patients underwent surgical treatment. Patients did not receive drug treatments before surgery and exhibited no symptoms of distant metastasis. Gastric samples were obtained from the seven healthy volunteers. The tissues were isolated and frozen at −80°C instantly immediately after the surgery. Before implementation of the study, the Ethical Committee of the Second Hospital of Dalian Medical University approved the study protocol. All patients provided informed consent. Diagnoses of the samples were performed by two independent pathologists.

**TABLE 1:** Characteristics of patients with gastric cancer and healthy volunteers.

Group	Patients (*n* = 33)	Healthy volunteers (*n* = 7)
**Sex**		
Male	19	4
Female	14	3
**Age (years)**		
>50	21	5
≤50	12	2
**Tumor size (cm)**		
>5	9	
≤5	24	
**TNMstage**		
I–II	15	
III–IV	18	
**Distant metastasis**		
Negative	14	
Positive	19	
**Lymphatic metastasis**		
No	25	
Yes	8	

### Cell Culture

GC cell lines SGC-7901, MKN-28, MKN-45, NCI-N87, and BGC-823 were provided by Shanghai Institutes for Biological Sciences, Chinese Academy of Sciences. The immortalized healthy GES-1 was a gift from Professor Yan Min (Shanghai Jiao Tong University). The cells were cultivated in Roswell Park Memorial Institute-1640 containing thermally inactivated fetal bovine serum (10%), penicillin (100 U/ml), and streptomycin (0.1 mg/ml) under a damp 5% CO_2_ atmosphere at 37°C. Cells were selected for subsequent experiments at exponential growth phrases.

### Cell Transfection

After being inoculated in 6-well plates (2 × 10^5^ cells/well), cells were subjected to negative control (NC) small hairpin RNA (shRNA), SNHG11 shRNA, miR-184 mimic, NC mimic, miR-184 inhibitor, or NC inhibitor, with Lipofectamine 2000 (Thermo Fisher Scientific, Waltham, MA), when the cell confluence was 80%. The medium was replaced 6 h after transfection, and cells were collected approximately 1.5–2 days after transfection.

### Lentiviral Infection

After being phosphorylated and annealed, the lncRNA SNHG11 shRNA oligonucleotides and the scrambled NC shRNA sequence were cloned to pLVX-puro vector (Clontech), designating as pLVX-siSNHG11 and pLVX-siNC. Lentiviral-siSNHG11 and lentiviral-siNC particles were prepared by triple 293T cell transfection (Invitrogen, Carlsbad, CA) with vectors pLVX-siSNHG11 and pLVX-siNC, respectively, as well as pMD2.G and psPAX2. For infection, SGC-7901 cells were cultivated with polybrene (5 μg/ml) and lentiviral particles. Then, 6 h later, the medium was removed, and the cells were connected for subsequent experiments.

### Quantitative Polymerase Chain Reaction

Total RNA (2 μg) was separated from GC cell lines and specimens (0.1 g) with TRIzol (Invitrogen), and its concentrations were determined using Nanodrop2000 (OD260) (Thermo Fisher Scientific). cDNA was prepared through reverse transcription via Oligo (dT) 20 primer (Thermo Fisher Scientific) and MMLV First-Strand Kit (Invitrogen) for quantitative polymerase chain reaction (qPCR). Gene qPCR detection was performed using the SYBR^®^ Green dye (Takara) detection method, and all procedures were conducted in accordance with the manufacturer’s instructions. The reaction system was prepared by first denaturation for 10 min at 95°C, 40 denaturation cycles for 15 s at 95°C, and an extension for 40 s at 60°C. The relative gene expression was defined using the following equation: ΔCt = Ct_target_-Ct_reference_. The fold-change for target genes normalized by U6 and GAPDH was determined using the 2^–ΔΔCt^ method. All experiments were conducted three times in parallel.

### Western Blotting

Cells were lysed through the cultivation of cells with radioimmunoprecipitation assay buffer (pH, 8.0) and a protease inhibitor cocktail (Roche Applied Science, Penzberg, Germany). Protein concentrations were determined using a Bicinchoninic Acid Assay kit. Protein was subsequently presented with sodium dodecyl sulfate-polyacrylamide gel electrophoresis and added electrically to polyvinylidene fluoride membranes (Millipore, MA). The available sites were cultivated overnight at 4°C with primary antibodies and were then washed with tris-buffered saline. Next, Western blotting was detected by incubating for 60 min at room temperature with secondary antibodies. After being washed with tris-buffered saline multiple times, the bands on the membranes were visualized using a Maximum Sensitivity Substrate Kit (Thermo Fisher Scientific).

### Cell Proliferation

After proliferation assessment using a cell counting kit-8 (CCK-8) assay (Li et al., 2014) as for relevant guidance, cells were inoculated into 96-well plates. Then, the medium containing CCK-8 (0.01 ml) was transferred into each well, and the cells were cultivated for another 120 min at 37°C. The optical density (OD_450_) was determined using the Infinite M200 (Tecan, Männedorf, Switzerland).

### Colony Formation Assay

A single-cell suspension was prepared for counting with 500 cells in a 0.3-cm culture medium. After being cultured for 4 days, cell specimens in phosphate buffered saline were collected and fixed with methanol before being subjected to crystal violet staining. A clone number of >50 cells was measured through a microscope (Olympus Corp., Tokyo, Japan) (Deng et al., 2016).

### Transwell Migration Assay

Cells were presented with trypsinization and rinsed one time with D-Hanks solution. To determine cell migration or intrusion, pore size (8 μm) culture or Matrigel inserts were placed in 24-well plates. Then, 0.4 mL of F-12 with hepatocyte growth factor (0.02 μg/ml) and fetal bovine serum (10%) were supplemented to the lower chamber. Next, 1 × 10^5^ cells were added into the upper chamber. After being cultivated for 20 h, the cells that went through the pores were identified with CV staining and observed through a microscope (Li et al., 2014).

### Wound-Healing Assay

Confluent cells were scraped through a pipette tip (10 μL). The cells migrated into the wound and were fixed. Afterward, the scratched area was observed through a microscope. The percentage (%) of migration percentage was obtained using the formula: width_2__d_/width_0__d_.

### Bioinformatics

We used the prediction algorithms starBase^1^ and TargetScan^2^ to identify the targets of SNHG11 and miR-184, respectively. Predictions are listed as the targeting prediction efficacy. In addition, predictions are listed based on their conserved targeting possibility (Friedman et al., 2009).

### Dual-Luciferase Reporter Assay

Dual-luciferase reporter assay was performed, as described previously (Wang et al., 2018). Briefly, after amplification through polymerase chain reaction, part of the DNA sequences of CDC25A or lncRNA SNHG11 with mutant (MU) or wild-type (WT) miR-184 binding sites were subsequently cloned to a pmirGLO dual-luciferase plasmid (Promega, Madison, WI) to prepare SNHG11-WT, SNHG11-MU, CDC25A-WT, and CDC25A-MU reporter plasmids that were individually transfected with miR-184 or NC mimics into HEK293T cells. When completed, the luciferase activities were assessed using the dual-luciferase reporter assay system (Promega).

### Xenograft Tumors in Nude Mice

The *in vivo* tumor growth experiment was performed, as described previously (Li et al., 2014). Briefly, eight clean grade female Balb/c nude mice (age, 28–36 days; weight, 20 ± 2 g) were provided by the Animal Experimental Center of Dalian Medical University. SGC-7901 cells transduced with lentivirus with siNC or siSNHG11 were resuspended in Matrigel (50%; BD Biosciences, Bedford, MA). The cell concentration was 1 × 10^7^ cells/mL. Then, a single-cell suspension (0.2 mL, 5 × 10^6^ cells) was injected into the mice through the left axilla. Each group included eight mice. At day 30, the mice were sacrificed, and the tumor weight, volume, and lymph node metastasis were evaluated.

### Data Analysis

All data were presented as average ± standard deviation. One-way analysis of variance was applied to analyze the distinctions between multiple groups; *t*-tests were used for comparing the two groups. A *P* < 0.05 was considered as significant.

## Results

### MiR-184 Linked SNHG11 and CDC25A

**FIGURE 1:**
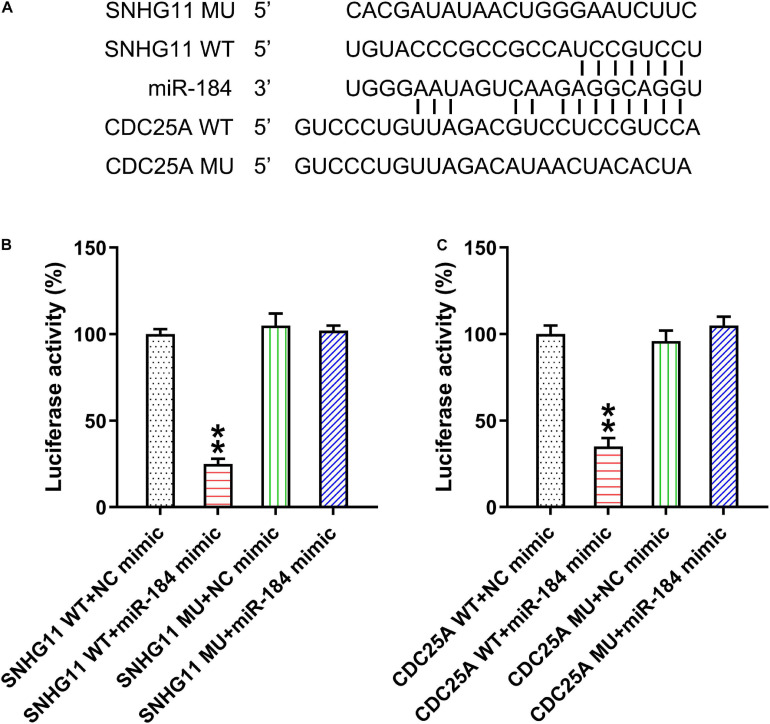
LncRNA SNHG11 targeted miR-184 and miR-184 targeted CDC25A. **(A)** Illustration of the conserved SNHG11-binding motifs in miR-184, and the conserved miR-184-binding motifs in CDC25A 3′-UTR. **(B,C)** Luciferase functions were assessed with luciferase reporter products consisting of WT or MU copy of SNHG11 and CDC25A after miR-184 mimic transfection in HEK293T cells with Renilla luciferase as a reference. These data are shown as average ± *SD*. **P < 0.01 vs. indicated groups (hereinafter similarly).

Several studies have demonstrated that lncRNA SNHG11 serves as a miR-184 sponge in hepatocellular cancer cells (Huang et al., 2020), whereas the latter targeted 3′-UTR of CDC25A in non-small cell lung cancer (Lin et al., 2015). Thus, we considered whether miR-184 linked SNHG11 and CDC25A. Bioinformatics was therefore conducted to examine the targets of SNHG11 and miR-184. Bioinformatics clearly demonstrated that SNHG11 targets miR-184 and that the latter could target the 3′-UTR of CDC25A (Figure 1A). Dual-luciferase reporter assay was then conducted in the HEK293T cells to confirm the mechanistic correlations between SNHG11-miR-184 and miR-184-CDC25A (Figures 1B,C).

### Correlation of lncRNA SNHG11, miR-184, and CDC25A in GC Specimens and Cell Lines

**FIGURE 2:**
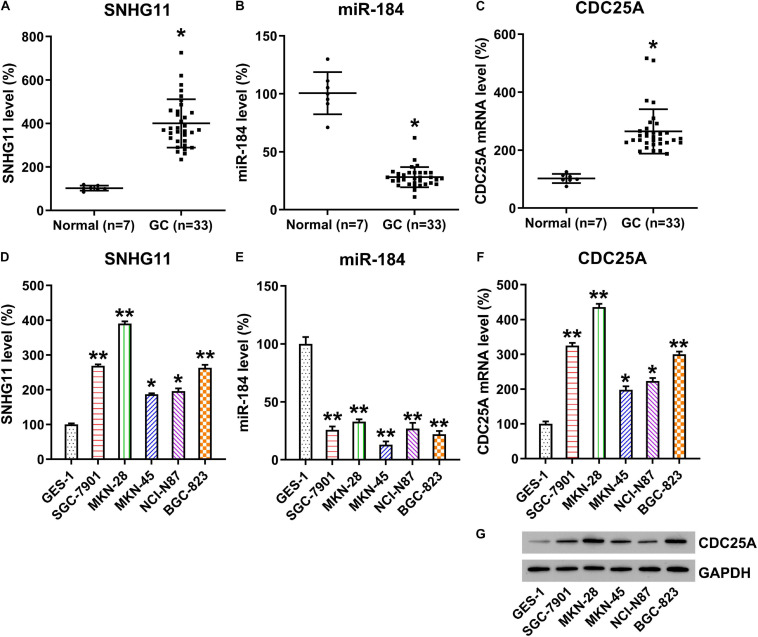
Expressions of SNHG11, miR-184, and CDC25A in GC specimens and cell lines. **(A–C)** qPCR detection showed expressions of SNHG11, miR-184, and CDC25A in GC specimens (*n* = 33) and in specimens from healthy gastric tissue (*n* = 7). **(D–F)** qPCR analysis showed expression levels of HCG11, miR-184, and CDC25A in GC and GES-1 cell lines. **(G)** Western blotting analysis showed expression levels of CDC25A in GC and GES-1 cells. These data are shown as average ± *SD*. *P < 0.05, **P < 0.01 vs. indicated groups.

To reveal the role of SNHG11, miR-184, and CDC25A during GC development, we analyzed SNHG11, miR-184, and CDC25A expression by qPCR in 33 GC samples and seven healthy normal gastric tissues. Compared with normal controls, SNHG11 expression and CDC25A was significantly upregulated, whereas the miR-184 level was downregulated in GC samples (Figures 2A–C). Moreover, we also found that SNHG11 and CDC25A were also increased in GC cell lines, whereas miR-184 expression was downregulated compared with GES-1 cells (Figures 2D–G), suggesting that SNHG11, miR-184, and CDC25A play a role in GC development.

**FIGURE 3:**
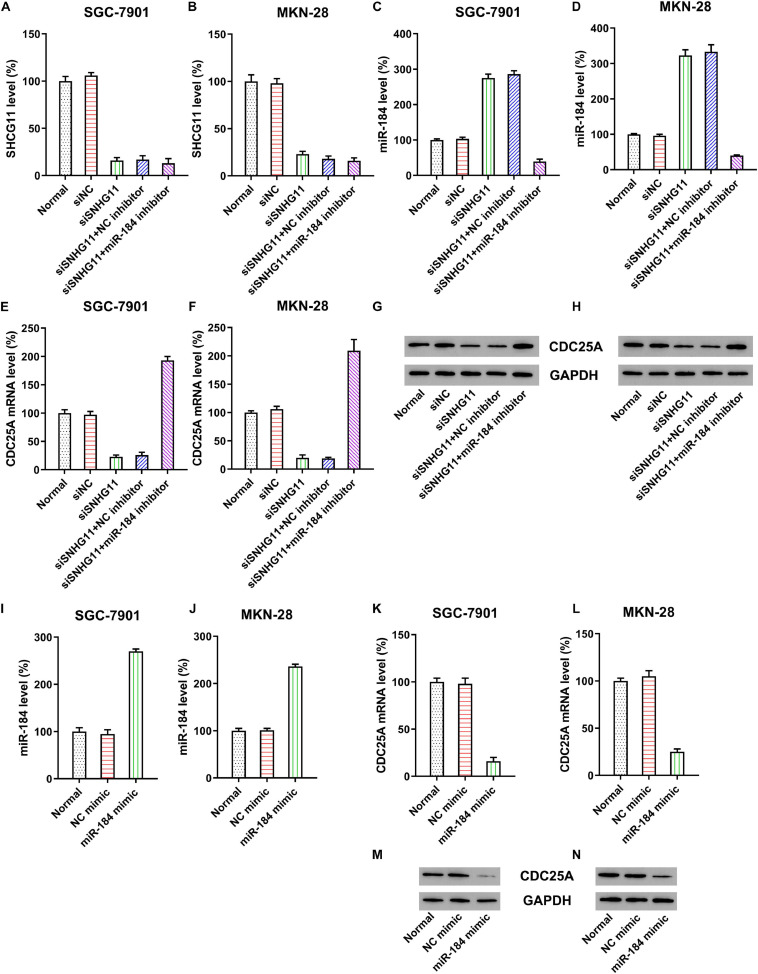
The correlation of SNHG11, miR-184, and CDC25A expression in MKN-28 and SGC-7901 cells. MKN-28 and SGC-7901 cells were subjected to NC shRNA, SNHG11 shRNA, SNHG11 shRNA + NC inhibitor, SNHG11 shRNA + miR-184 inhibitor for 36 h. qPCR assays were used to indicate the expression of **(A,B)** SNHG11, **(C,D)** miR-184, and **(E,F)** CDC25A mRNA in cells. **(G,H)** Western blotting analysis was conducted to indicate the CDC25A protein expression in cells treated with NC mimic, miR-184 mimic for 36 h, or not. qPCR assays were used to indicate the expression of **(I,J)** miR-184, **(K,L)** CDC25A mRNA in cells. **(M,N)** Western blotting analysis was conducted to indicate the CDC25A protein expression in cells. “Normal” represents non-transfected cells.

To further probe the relationship between SNHG11, miR-184, and CDC25A, MKN-28, and SGC-7901 were transfected with SNHG11 shRNA or NC shRNA. qPCR data confirmed that the SNHG11 level was significantly decreased through SNHG11 shRNA transfection (Figures 3A,B). Furthermore, qPCR also indicated that transfection with SNHG11 shRNA caused a significant increase in miR-184 (Figures 3C,D) and decreased CDC25A (Figures 3E–H) compared with the siNC group. MKN-28 and SGC-7901 cells were then subjected to SNHG11 shRNA and miR-184 or NC inhibitor to assess the role of miR-184 in SNHG11-mediated CDC25A expression. miR-184 inhibition resulted in an obvious reduced miR-184 level in cells with SNHG11 knockdown (Figures 3C,D). Moreover, CDC25A mRNA and protein expressions were obviously decreased after miR-184 inhibition (Figures 3E–H).

To assess the direct correlation of CDC25A and miR-184, cells were subjected to miR-184 or NC mimic. Compared with the NC mimic group, miR-184 transfection dramatically improved the miR-184 expression in MKN-28 and SGC-7901 cells (Figures 3I,J). Cells transfected with miR-184 mimic showed decreased expression of CDC25A at mRNA and protein levels (Figures 3K–N).

These data clearly suggest that CDC25A expression was negatively regulated by miR-184, whereas the latter was sponged by SNHG11. Therefore, CDC25A expression was positively correlated to lncRNA SNHG11 in GC cells.

### SNHG11 Knockdown and miR-184 Upregulation Suppressed GC Cell Growth and Proliferation

**FIGURE 4:**
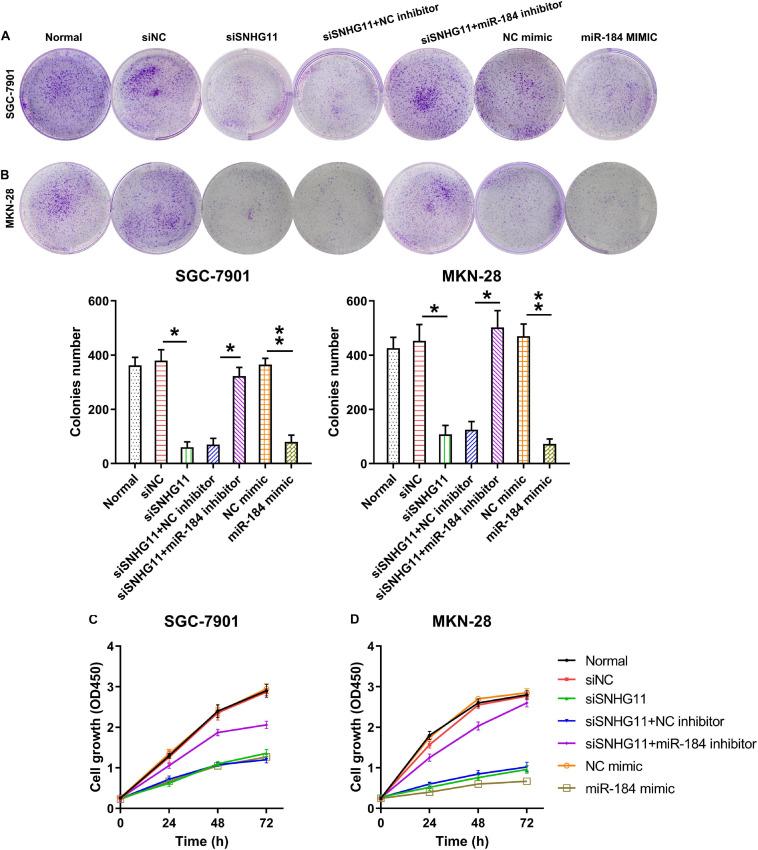
The part of SNHG11 and miR-184 in MKN-28 and SGC-7901cells’ growth and proliferation. MKN-28 and SGC-7901 cells were subjected to NC shRNA, SNHG11 shRNA, SNHG11 shRNA + NC inhibitor, SNHG11 shRNA + miR-184 inhibitor, NC mimic, and miR-184 mimic for 36 h, or not. **(A,B)** Soft agar colony generation assay for cells at 96 h after transfection. **(C,D)** CCK-8 assay demonstrated cell proliferation at 1, 2, and 3 days after transfection. “Normal” represents non-transfected cells. These data are shown as average ± *SD*. *P < 0.05, **P < 0.01 vs. indicated groups.

The roles of SNHG11 and miR-184 in MKN-28 and SGC-7901 cell growth and proliferation were investigated by colony formation and CCK-8 assay. Our colony formation experiment demonstrated that SNHG11 knockdown and miR-184 elevation significantly decreased the number of colonies formed by MKN-28 and SGC-7901 cells, whereas miR-184 inhibition caused a significant recovery in the growth of the two GC cells with SNHG11 silencing compared with SNHG11 silencing and NC inhibitor group (Figures 4A,B).

The CCK-8 assay was then conducted to detect cell proliferation at 1, 2, and 3 days after transfection. The data demonstrated that the proliferation rates were dramatically reduced at 1, 2, and 2 days after transfection through SNHG11 silencing or miR-184 overexpression compared with that in the siNC or NC mimic groups, respectively. In addition, miR-184 inhibition in cells with SNHG11 knockdown was able to restore the cell proliferation to a normal level (Figures 4C,D). These data suggested a regulatory role of SNHG11-miR-184 axis in colorectal cancer cell proliferation.

### SNHG11 Knockdown and miR-184 Upregulation Induced GC Cell Apoptosis

**FIGURE 5:**
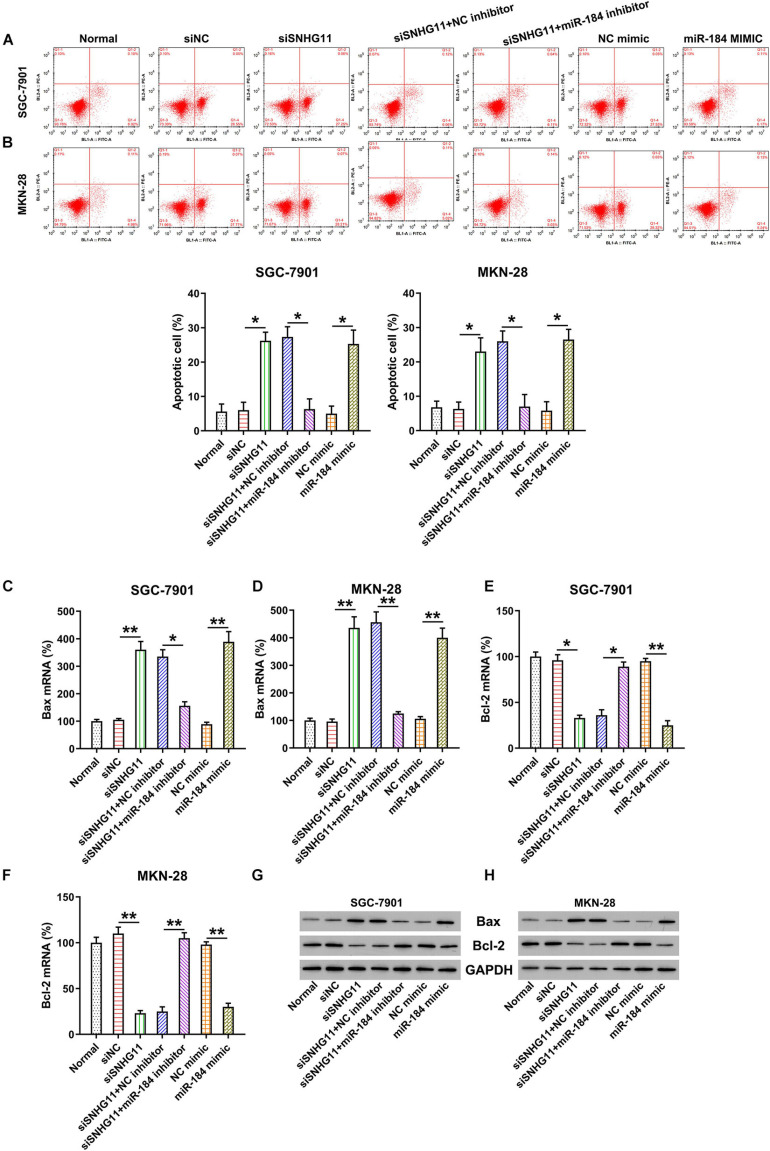
The effect of SNHG11 and miR-184 on MKN-28 and SGC-7901 cells’ apoptosis. MKN-28 and SGC-7901 cells were subjected to NC shRNA, SNHG11 shRNA, SNHG11 shRNA + NC inhibitor, SNHG11 shRNA + miR-184 inhibitor, NC mimic, and miR-184 mimic for 36 h, or not. **(A,B)** Annexin V-FITC/PI FC results demonstrated the number of apoptotic cells. **(C–F)** qPCR assay and **(G,H)** Western blotting analysis were used to indicate the Bcl-2 and Bax mRNA and protein expressions in cells. “Normal” represents non-transfected cells. These data are shown as average ± *SD*. *P < 0.05, **P < 0.01 vs. indicated groups.

Given the evidence that SNHG11 knockdown and miR-184 upregulation decreased the MKN-28 and SGC-7901 cell proliferation, we hypothesized that SNHG11 regulates apoptosis of the GC cells. Therefore, Annexin V-FITC/PI flow cytometry (FC) and Bcl-2/Bax expression by qPCR and Western blotting analysis were used to examine the role of SNHG11 and miR-184 on apoptosis. Compared with the siNC and NC mimic groups, an obvious increment of apoptotic cell number were observed in the two GC cells transfected with the SNHG11 shRNA and miR-184 mimic (Figures 5A,B). Moreover, miR-184 inhibition reversed the effect of SNHG11 knockdown on the induction of apoptotic cell numbers (Figures 5A,B).

The role of SNHG11 silencing in Bcl-2 and Bax expressions was also analyzed by qPCR and Western blotting analysis. The Bcl-2 mRNA and protein expressions were obviously reduced in the SNHG11 silencing and miR-184 mimic groups (Figures 5C,D,G,H). However, the proapoptotic Bax mRNA and protein were promoted under SNHG11 knockdown and miR-184 upregulation (Figures 5E–H) in MKN-28 and SGC-7901 cells compared with those in the siNC and NC mimic groups, respectively. Notably, miR-184 inhibition counteracted the role of SNHG11 on Bcl-2/Bax expression, suggesting that miR-184 is involved in the SNHG11 knockdown-mediated apoptosis of GC cells.

### SNHG11 Knockdown and miR-184 Upregulation Reduced GC Cell Migration

**FIGURE 6:**
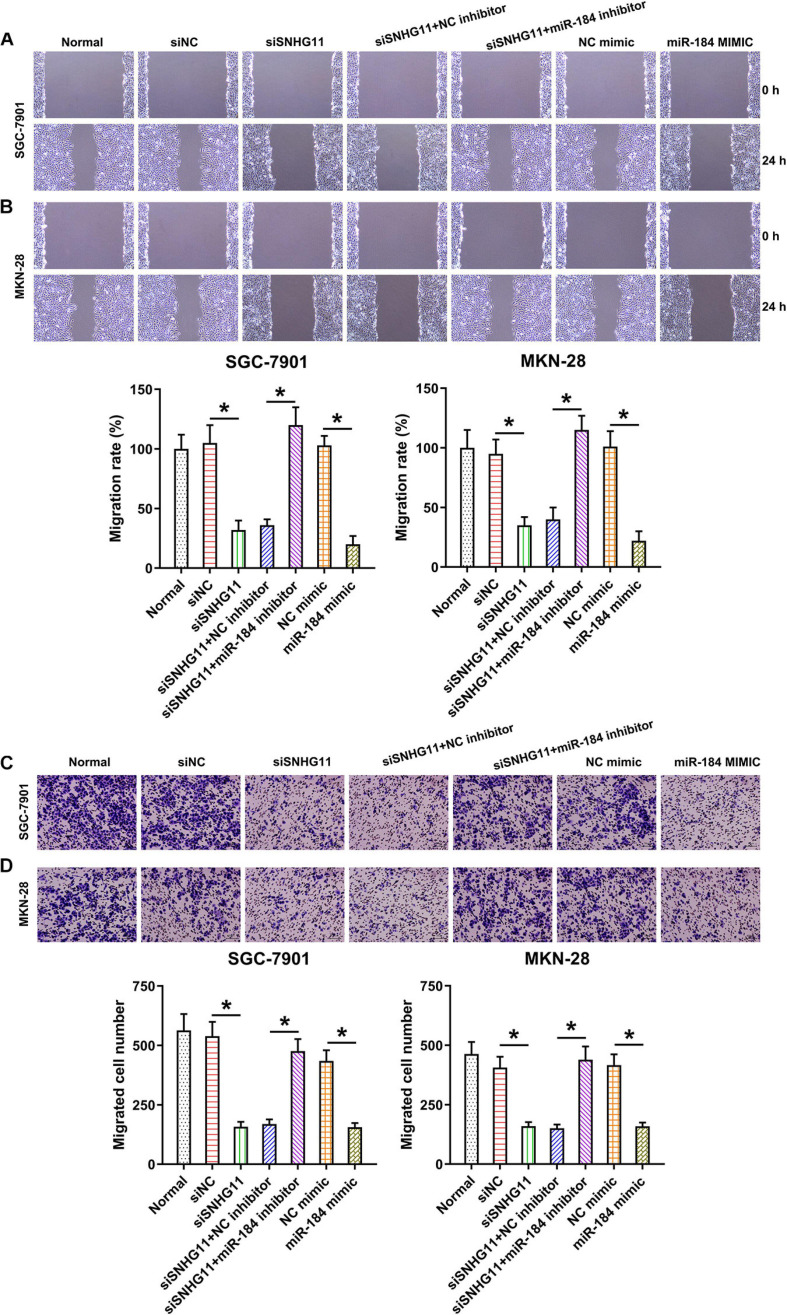
The effect of SNHG11 and miR-184 on MKN-28 and SGC-7901 cells’ migration and intrusion. MKN-28 and SGC-7901 cells were subjected to NC shRNA, SNHG11 shRNA, SNHG11 shRNA + NC inhibitor, SNHG11 shRNA + miR-184 inhibitor, NC mimic, and miR-184 mimic for 36 h, or not. **(A,B)** Migration and **(C,D)** intrusion capacity was measured by WHA and TMA. “Normal” represents non-transfected cells. These data are shown as average ± *SD*. *P < 0.05 vs. indicated groups.

To detect the role of SNHG11 and miR-184 in migration, wound-healing assay and Transwell migration assay were conducted. We found that SNHG11 silencing and miR-184 upregulation significantly decreased the migration rate of MKN-28 and SGC-7901 cells using wound-healing assay (Figures 6A,B). We also found that SNHG11 silencing and miR-184 upregulation inhibited cell intrusion capacity, as shown by Transwell migration assay (Figures 6C,D). Meanwhile, the inhibition of miR-184 also led to a noticeable restoration in the number of migrated and invasive MKN-28 and SGC-7901 cells, as evidence by wound-healing assay and Transwell assay (Figures 6A–D). Thus, these data suggested that SNHG11 and miR-184 axis influence SGC-7901 and MKN-28 cell migration by regulating the miR-184 level.

### SNHG11 Knockdown Inhibited Tumor Growth of GC in Nude Mice

**FIGURE 7:**
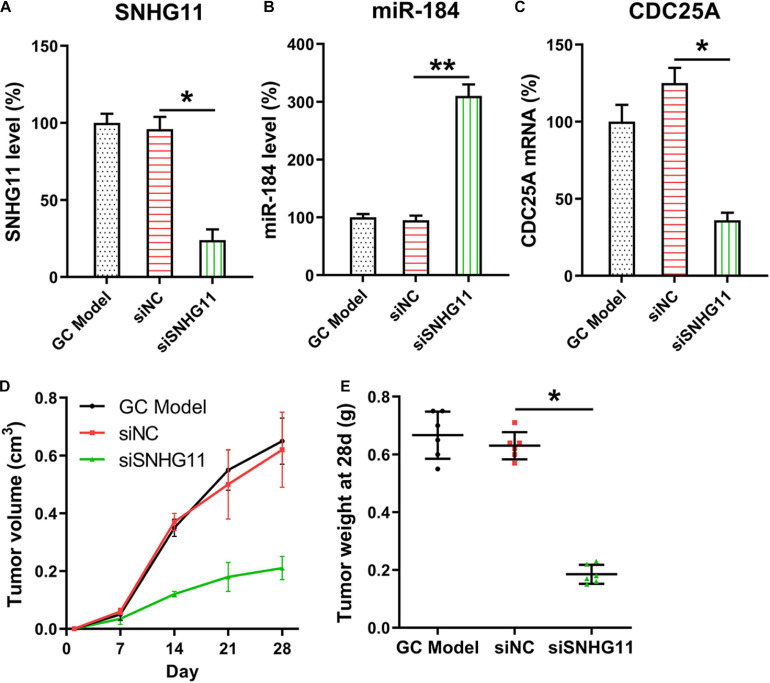
SNHG11 silencing inhibits xenograft GC tumorigenesis. The SGC-7901, SGC-7901-siNC and SGC-7901-siSNHG11 cells were injected through the right back flank of 6 week-old BALB/c-nu mice (*n* = 6). **(A)** SNHG11, **(B)** miR-184, and **(C)** CDC25A mRNA expressions in tumor specimens were determined through qPCR. **(D,E)** Mice were sacrificed, and the tumors were weighed 4 weeks after inoculation. Tumor weight and volume were measured. These data are shown as average ± *SD*. *P < 0.05, **P < 0.01 vs. indicated groups.

The mice were subcutaneously injected with SGC-7901, SGC-7901-siNC, and SGC-7901-siSNHG11 cells for examining the role of SNHG11 in xenograft GC tumor occurrence. The expressions of SNHG11, miR-184, and CDC25A mRNA in the tumor specimens (*n* = 6) of mice were detected. SNHG11 and CDC25A mRNA appeared to be downregulated, whereas miR-184 expression was elevated in the SGC-7901-siSNHG11 inoculated mice compared with mice in the siNC group (Figures 7A–C). Mice were sacrificed after 4 weeks, and the tumor was excised and weighed. These findings indicated that tumors in the siSNHG11 group underwent growth at a slower rate, as represented by lower mean tumor weight and volume, than those in the siNC group (Figures 7D,E).

## Discussion

GC threatens human health. Therefore, it is critical to investigate GC pathogenesis and discover potent early diagnosis indicators and biological treatment targets. Accumulating evidence reveals that lncRNA is highly associated with GC development and progression (Li et al., 2016). Previous studies have reported that lncRNA SNHG11 exhibited high expression levels in HCC and cell lines. In our study, qPCR demonstrated that SNHG11 expression was also ectopically upregulated in the GC specimens and cell lines compared with that in their healthy counterparts. Furthermore, we discovered that SNHG11 facilitates GC cell proliferation and migration through competitively connecting with miR-184, which subsequently upregulates the CDC25A expression.

LncRNA SNHGs have been extensively investigated in different tumors. Cai et al. (2017) reported that SNHG16 improves breast cancer cell migration by competitively connecting with E2F5 and miR-98, which possibly results in an unsatisfactory prognosis. Cao et al. indicated that SNHG1 serves as a miR-7 competing endogenous RNA to increase the NLRP3 level, thereby activating NLRP3 inflammasome in Parkinson’s disease (PD) (Cao et al., 2018). The SNHG1 expression was elevated in LPS-induced BV2 cells in an *in vitro* PD model. The upregulation of SNHG1 was elevated, and SNHG1 knockdown suppressed LPS-induced BV2 microglial activation and inflammation. Furthermore, SNHG1 functioned as a competing endogenous RNA for miR-7 to regulate nod-like receptor protein 3 (NLRP3) expression, leading to the activation of NLRP3 inflammasome. In the microglial culture supernatant transfer model, SNHG1 knockdown in LPS-stimulated BV2 cells inhibited primary neurons from apoptosis and elevated caspase-3 activity. Additionally, SNHG1 was increased in MPTP-induced PD mouse models. SNHG1 downregulation suppressed the activation of microglia and NLRP3 inflammasome as well as dopaminergic neuron loss in the midbrain substantia nigra pars compacta in MPTP-treated mice (Cao et al., 2018). LncRNA SNHG12 modulates GC progression by functioning as a molecular sponge of miR-320 (Zhang and Lu, 2018) and miR-199a/b-5p (Yang et al., 2018). Patients with high SNHG12 expression had a short survival period. Additionally, the inhibition of SNHG12 in GC cell lines SGC-7901 and AGS suppressed cell growth, colony formation, proliferation, and invasion (Yang et al., 2018). LncRNA SNHG11 has also been often reported as an oncogene in cancers. Although study regarding the correlation of SNHG11 and GC has been limited, their functions in other cancers have been affirmed. In prostate cancer, lncRNAs, including LINC00476, MALAT1, SNHG11, LINC00649, and ILF3-AS1, have the most nodes and higher betweenness centrality values, and are regarded as prognosis markers (Ye et al., 2019). In colorectal cancer, the marriage of LINC00909, LINC00654, ZFAS1, and SNHG11, demonstrated high diagnosis effects, especially in early-stage disease. A high SNHG11 level facilitates colorectal cancer cell proliferation and metastasis by acting on the Hippo pathway (Xu et al., 2019). In lung cancer, SNHG11 was increased and promoted cell proliferation, migration, intrusion, and epithelial–mesenchymal transition, whereas it inhibited apoptosis. Furthermore, a high SNHG11 level was relevant to unfavorable prognosis, tumor node metastasis stage, and tumor dimension. Several studies indicated that SNHG11 acts on lung cancer cells through the miR-4436a/Wnt/β-catenin pathway (Liu et al., 2020). In this study, SNHG11 was increased in GC GC specimens and cell lines. SNHG11 knockdown increased miR-184 and CDC25A expressions, thereby modulating cell vitality and biological processes. Current studies have demonstrated that CDC25A, a bispecific protein phosphatase, is an important cell cycle modulator, which counteracts the suppressive phosphorylation in CDKs and improves the activities of CDKs that cause cell cycle progression. In addition, CDC25A could also modulate apoptosis. Cdc25A overexpression promotes tumor occurrence and is frequently detected in diverse cancers (Shen and Huang, 2012). CDC25A expression was dramatically elevated in GC. Additionally, CDC25A overexpression promoted cell proliferation, migration, and intrusion, whereas it suppressed apoptosis (Yu et al., 2017).

Our data confirmed that a reduction in CDC25A expression resulting from an miR-184 increase is observed through a reduction in CDC25A mRNA instability because its coding area is targeted. Nowadays, a reduction in CDC25A because of miR-184 was also confirmed by acting on its coding area (Yu et al., 2017). The relevant action mechanism is different from that of miR-21, 15a, and 483-3p with reference to acting on a CDC25A 3′-untranslated region (Lee et al., 2008; Wang et al., 2009; Bertero et al., 2013). An elevation in miR-184 by SNHG11 knockdown also induced CDC25A ablation, whereas the inhibition of miR-184 in SNHG11 silenced cells showed higher expressed CDC25A, suggesting that CDC25A expression is positively correlated with SNHG11 level in GC cells. Hence, miR-184 can be considered as an antitumor miR, which represses GC progression by targeting CDC25A.

One of limitations of this study is that the correlations between SNHG11 and miR-184, miR-184, and CDC25A have already been reported in other types of cancers, such as lung cancer and hepatic cancer (Lin et al., 2015; Huang et al., 2020). This study combined the two findings and testified them in GC. This work confirmed that SNHG11 is increased in GC specimens and cell lines and demonstrated that it is negatively correlated with miR-184 and positively related to CDC25A. Moreover, the SNHG11/miR-184/CDC25A regulatory loop is important for GC cell proliferation, migration, and apoptosis. SNHG11 may provide a new biomarker for GC diagnosis, treatment, and prognosis.

## Data Availability Statement

The original contributions presented in the study are included in the article/supplementary material, further inquiries can be directed to the corresponding author/s.

## Ethics Statement

The operations conducted as part of this study were approved by the Ethics Committee of the Second Hospital of Dalian Medical University. All patients provided written informed consent before beginning of the study. Every procedure was approved by the Animal Care and Use Committee of The Second Hospital of Dalian Medical University. All possible measures were taken to decrease animal suffering as much as possible.

## Author Contributions

DZ and BW conceived the project. DZ, HC, and BW designed and performed the experiments. HC and BW analyzed the data. DZ wrote and revised the manuscript. All authors contributed to the article and approved the submitted version.

## Conflict of Interest

The authors declare that the research was conducted in the absence of any commercial or financial relationships that could be construed as a potential conflict of interest.

## Footnotes

1 http://starbase.sysu.edu.cn/

2 http://www.targetscan.org

## Abbreviations

CCK-8: cell counting kit-8
FC: flow cytometry
GC: gastric cancer
HCC: hepatocellular cancer
lncRNAs: long non-coding RNAs
MU: mutant
NC: negative control
ncRNAs: non-coding RNAs
miR-184: microRNA-184
qPCR: quantitative polymerase chain reaction
shRNA: small hairpin RNA
WT: wild-type.

## References

[B1] Adams B. D. Anastasiadou E. Esteller M. He L. Slack F. J. (2015). *The Inescapable Influence of Noncoding RNAs in Cancer.* Philadelphia: AACR. 10.1158/0008-5472.CAN-15-1989 PMC4823153 26567137

[B2] Bertero T. Gastaldi C. Bourget-Ponzio I. Mari B. Meneguzzi G. Barbry P. (2013). CDC25A targeting by miR-483-3p decreases CCND–CDK4/6 assembly and contributes to cell cycle arrest. *Cell Death Differ.* 20 800–811. 10.1038/cdd.2013.5 23429262 PMC3647239

[B3] Cai C. Huo Q. Wang X. Chen B. Yang Q. (2017). SNHG16 contributes to breast cancer cell migration by competitively binding miR-98 with E2F5. *Biochem. Biophys. Res. Commun.* 485 272–278. 10.1016/j.bbrc.2017.02.094 28232182

[B4] Cancer Genome Atlas Research Network (2014). Comprehensive molecular characterization of gastric adenocarcinoma. *Nature* 513 202–209. 10.1038/nature13480 25079317 PMC4170219

[B5] Cao B. Wang T. Qu Q. Kang T. Yang Q. (2018). Long noncoding RNA SNHG1 promotes neuroinflammation in Parkinson’s disease via regulating miR-7/NLRP3 pathway. *Neuroscience* 388 118–127. 10.1016/j.neuroscience.2018.07.019 30031125

[B6] Chen H. Ren C. Han C. Wang D. Chen Y. Fu D. (2015). Expression and prognostic value of miR-486-5p in patients with gastric adenocarcinoma. *PLoS One* 10:e0119384. 10.1371/journal.pone.0119384 25793394 PMC4368750

[B7] Deng W. Wang J. Zhang J. Cai J. Bai Z. Zhang Z. (2016). TET2 regulates LncRNA-ANRIL expression and inhibits the growth of human gastric cancer cells. *IUBMB Life* 68 355–364. 10.1002/iub.1490 27027260

[B8] Ferlay J. Shin H. R. Bray F. Forman D. Mathers C. Parkin D. M. (2010). Estimates of worldwide burden of cancer in 2008: GLOBOCAN 2008. *Int. J. Cancer* 127 2893–2917. 10.1002/ijc.25516 21351269

[B9] Friedman R. C. Farh K. K.-H. Burge C. B. Bartel D. P. (2009). Most mammalian mRNAs are conserved targets of microRNAs. *Genome Res.* 19 92–105. 10.1101/gr.082701.108 18955434 PMC2612969

[B10] Huang W. Huang F. Lei Z. Luo H. (2020). LncRNA SNHG11 promotes proliferation, migration, apoptosis, and autophagy by regulating hsa-miR-184/AGO2 in HCC. *OncoTargets Ther.* 13 413–421. 10.2147/ott.s237161 32021286 PMC6969695

[B11] Kolata G. (2012). “Bits of mystery DNA, far from ‘junk,’play crucial role,” in *The New York Times*, September 18, 1851.

[B12] Lee S.-O. Masyuk T. Splinter P. Banales J. M. Masyuk A. Stroope A. (2008). MicroRNA15a modulates expression of the cell-cycle regulator Cdc25A and affects hepatic cystogenesis in a rat model of polycystic kidney disease. *J. Clin. Invest.* 118 3714–3724. 10.1172/jci34922 18949056 PMC2571032

[B13] Li H. Yu B. Li J. Su L. Yan M. Zhu Z. (2014). Overexpression of lncRNA H19 enhances carcinogenesis and metastasis of gastric cancer. *Oncotarget* 5 2318–2329. 10.18632/oncotarget.1913 24810858 PMC4039165

[B14] Li T. Mo X. Fu L. Xiao B. Guo J. (2016). Molecular mechanisms of long noncoding RNAs on gastric cancer. *Oncotarget* 7 8601–8612. 10.18632/oncotarget.6926 26788991 PMC4890990

[B15] Lin T.-C. Lin P.-L. Cheng Y.-W. Wu T.-C. Chou M.-C. Chen C.-Y. (2015). MicroRNA-184 deregulated by the microRNA-21 promotes tumor malignancy and poor outcomes in non-small cell lung cancer via targeting CDC25A and c-Myc. *Ann. Surg. Oncol.* 22 1532–1539. 10.1245/s10434-015-4595-z 25990966

[B16] Liu S. Yang N. Wang L. Wei B. Chen J. Gao Y. (2020). lncRNA SNHG11 promotes lung cancer cell proliferation and migration via activation of Wnt/β-catenin signaling pathway. *J. Cell. Physiol.* 235 7541–7553. 10.1002/jcp.29656 32239719

[B17] Malzkorn B. Wolter M. Liesenberg F. Grzendowski M. Stühler K. Meyer H. E. (2010). Identification and functional characterization of microRNAs involved in the malignant progression of gliomas. *Brain Pathol.* 20 539–550. 10.1111/j.1750-3639.2009.00328.x 19775293 PMC8094849

[B18] Pant T. Dhanasekaran A. Bai X. Zhao M. Thorp E. B. Forbess J. M. (2019). Genome-wide differential expression profiling of lncRNAs and mRNAs associated with early diabetic cardiomyopathy. *Sci. Rep.* 9:15345. 10.1038/s41598-019-51872-9 PMC6814824 31653946

[B19] Rochet E. Appukuttan B. Ma Y. Ashander L. M. Smith J. R. (2019). Expression of long non-coding RNAs by human retinal müller glial cells infected with clonal and exotic virulent toxoplasma gondii. *Noncoding RNA* 5:48. 10.3390/ncrna5040048 31547203 PMC6958423

[B20] Schwarzer A. Emmrich S. Schmidt F. Beck D. Ng M. Reimer C. (2017). The non-coding RNA landscape of human hematopoiesis and leukemia. *Nat. Commun.* 8:218. 10.1038/s41467-017-00212-4 PMC5550424 28794406

[B21] Shen T. Huang S. (2012). The role of Cdc25A in the regulation of cell proliferation and apoptosis. *Anticancer Agents Med. Chem.* 12 631–639. 10.2174/187152012800617678 22263797 PMC3544488

[B22] Tivnan A. Foley N. H. Tracey L. Davidoff A. M. Stallings R. L. (2010). MicroRNA-184-mediated inhibition of tumour growth in an orthotopic murine model of neuroblastoma. *Anticancer Res.* 30 4391–4395. 21115884 PMC5030819

[B23] Wadhwa R. Song S. Lee J.-S. Yao Y. Wei Q. Ajani J. A. (2013). Gastric cancer—molecular and clinical dimensions. *Nat. Rev. Clin. Oncol.* 10 643–655. 10.1038/nrclinonc.2013.170 24061039 PMC3927982

[B24] Wang J. Yang X. Li R. Wang L. Gu Y. Zhao Y. (2018). Long non-coding RNA MYU promotes prostate cancer proliferation by mediating the miR-184/c-Myc axis. *Oncol. Rep.* 40 2814–2825. 30132573 10.3892/or.2018.6661

[B25] Wang P. Zou F. Zhang X. Li H. Dulak A. Tomko R. J. (2009). microRNA-21 negatively regulates Cdc25A and cell cycle progression in colon cancer cells. *Cancer Res.* 69 8157–8165. 10.1158/0008-5472.can-09-1996 19826040 PMC2763324

[B26] Xin Y. He X. Zhao W. Zhan M. Li Y. Xiao J. (2019). LncRNA PCAT6 increased cholangiocarcinoma cell proliferation and invasion via modulating miR-330-5p. *Am. J. Transl. Res.* 11 6185–6195. 31632586 PMC6789233

[B27] Xu W. Zhou G. Wang H. Liu Y. Chen B. Chen W. (2019). Circulating lncRNA SNHG11 as a novel biomarker for early diagnosis and prognosis of colorectal cancer. *Int. J. Cancer* 146 2901–2912. 10.1002/ijc.32747 31633800

[B28] Yang B. Cai W. Chen B. (2018). LncRNA SNHG12 regulated the proliferation of gastric carcinoma cell BGC-823 by targeting microRNA-199a/b-5p. *Eur. Rev. Med. Pharmacol. Sci.* 22 1297–1306. 29565487 10.26355/eurrev_201803_14471

[B29] Ye G. Guo L. Xing Y. Sun W. Yuan M. (2019). Identification of prognostic biomarkers of prostate cancer with long non-coding RNA-mediated competitive endogenous RNA network. *Exp. Ther. Med.* 17 3035–3040. 30906477 10.3892/etm.2019.7277 PMC6425256

[B30] Yu M. Xue Y. Zheng J. Liu X. Yu H. Liu L. (2017). Linc00152 promotes malignant progression of glioma stem cells by regulating miR-103a-3p/FEZF1/CDC25A pathway. *Mol. Cancer* 16:110. 10.1186/s12943-017-0677-9 PMC5485714 28651608

[B31] Zhang H. Lu W. (2018). LncRNA SNHG12 regulates gastric cancer progression by acting as a molecular sponge of miR-320. *Mol. Med. Rep.* 17 2743–2749. 29207106 10.3892/mmr.2017.8143

